# Bioavailable estradiol concentrations are elevated and predict mortality in septic patients: a prospective cohort study

**DOI:** 10.1186/s13054-016-1525-9

**Published:** 2016-10-21

**Authors:** Greg Tsang, Michael B. Insel, Justin M. Weis, Mary Anne M. Morgan, Michael S. Gough, Lauren M. Frasier, Cynthia M. Mack, Kathleen P. Doolin, Brian T. Graves, Michael J. Apostolakos, Anthony P. Pietropaoli

**Affiliations:** 1 Division of Pulmonary and Critical Care Medicine, Department of Medicine, University of Rochester Medical Center, 601 Elmwood Avenue, Rochester, NY 14642 USA; 2 Department of Nursing, University of Rochester Medical Center, 601 Elmwood Avenue, Rochester, NY 14642 USA; 3 College of Nursing, University of South Florida, MDC22, 12901 Bruce B. Downs Boulevard, Tampa, FL 33612 USA

**Keywords:** Estrogens, Estradiol, Sepsis, Septic shock, Sex, Critical care

## Abstract

**Background:**

Experimental studies demonstrate beneficial immunological and hemodynamic effects of estradiol in animal models of sepsis. This raises the question whether estradiol contributes to sex differences in the incidence and outcomes of sepsis in humans. Yet, total estradiol levels are elevated in sepsis patients, particularly nonsurvivors. Bioavailable estradiol concentrations have not previously been reported in septic patients. The bioavailable estradiol concentration accounts for aberrations in estradiol carrier protein concentrations that could produce discrepancies between total and bioavailable estradiol levels. We hypothesized that bioavailable estradiol levels are low in septic patients and sepsis nonsurvivors.

**Methods:**

We conducted a combined case-control and prospective cohort study. Venous blood samples were obtained from 131 critically ill septic patients in the medical and surgical intensive care units at the University of Rochester Medical Center and 51 control subjects without acute illness. Serum bioavailable estradiol concentrations were calculated using measurements of total estradiol, sex hormone-binding globulin, and albumin. Comparisons were made between patients with severe sepsis and control subjects and between hospital survivors and nonsurvivors. Multivariable logistic regression analysis was also performed.

**Results:**

Bioavailable estradiol concentrations were significantly higher in sepsis patients than in control subjects (211 [78–675] pM vs. 100 [78–142] pM, *p* < 0.01) and in sepsis nonsurvivors than in survivors (312 [164–918] pM vs. 167 [70–566] pM, *p* = 0.04). After adjustment for age and comorbidities, patients with bioavailable estradiol levels above the median value had significantly higher risk of hospital mortality (OR 4.27, 95 % CI 1.65–11.06, *p* = 0.003). Bioavailable estradiol levels were directly correlated with severity of illness and did not differ between men and women.

**Conclusions:**

Contrary to our hypothesis, bioavailable estradiol levels were elevated in sepsis patients, particularly nonsurvivors, and were independently associated with mortality. Whether estradiol’s effects are harmful, beneficial, or neutral in septic patients remains unknown, but our findings raise caution about estradiol’s therapeutic potential in this setting. Our findings do not provide an explanation for sex-based differences in sepsis incidence and outcomes.

**Electronic supplementary material:**

The online version of this article (doi:10.1186/s13054-016-1525-9) contains supplementary material, which is available to authorized users.

## Background

Epidemiological studies show that sex influences both the incidence and the outcomes of sepsis syndrome. While the incidence of sepsis appears to be higher in men [1], some studies paradoxically suggest that sepsis mortality may be higher in women [2, 3]. The mechanisms for these epidemiological differences are unclear, but potential explanations include sex differences in clinical illness presentation, care delivery, or biological factors, including possible effects of sex hormones [2–4].

Estrogens have protective effects in animal models of sepsis [5, 6]. For example, sepsis mortality was lower in proestrus female rats than in males and ovariectomized females [6] and than in rats receiving an estrogen receptor agonist [7]. These findings might be explained by several possible salutary effects of estrogen, including downregulation of proinflammatory cytokines [8–10], augmentation of vital organ blood flow [11], improved cardiac contractility [7], and lower production of reactive oxygen species [11]. In contrast, clinical studies demonstrate that estrogen concentrations are elevated in critically ill patients and that higher levels are associated with poor outcomes [12–14]. Interestingly, blood estrogen concentrations in critically ill patients are determined primarily by peripheral fat androgen aromatization [15] and do not differ significantly by sex [12, 14].

Most previous clinical studies of estrogen levels in critical illness have included a wide array of intensive care unit (ICU) patients with and without infection. Ideally, relationships between estrogen and sepsis should include an exclusively septic study group because the initial cytokine cascade and metabolic derangements of sepsis may be unique [16]. Angstwurm and colleagues focused on infected ICU patients and observed higher total estradiol levels in nonsurvivors than in survivors [14]. However, it is unknown whether this association between estradiol concentrations and mortality is confounded by potentially important covariables. Most importantly, sepsis-associated alterations in estradiol carrier protein concentrations could permit high total yet paradoxically low bioactive hormone concentrations in nonsurvivors. This is possible because circulating estradiol binds to albumin and sex hormone-binding globulin (SHBG). Hormone that is bound to SHBG is inactive because it is not readily accessible to target tissues [17]. Malnutrition is associated with elevated SHBG levels [18] and is a common feature of critical illness [19]. The bioavailable fraction of circulating total estradiol is the sum of the free, unbound, and albumin-bound fractions. Albumin concentrations decline in critical illness and are negatively correlated with patient mortality [20]. Therefore, if sepsis causes relatively low albumin and high SHBG concentrations, septic patients could have high total but low bioavailable estradiol levels. Such alterations in estradiol carrier proteins would confound the association between high total estradiol levels and mortality.

To our knowledge, concentrations of the bioavailable fraction of circulating estradiol have not been reported previously in septic patients. The aim of this study was to determine the bioavailable fractions of estradiol in blood obtained from septic ICU patients. We also aimed to evaluate relationships between bioavailable estradiol and mortality after adjusting for common clinical confounding variables. We hypothesized that bioavailable estradiol concentrations would be low in critically ill septic patients and that low bioavailable estradiol concentrations would be associated with higher mortality.

## Methods

### Study design

**Fig. 1:**
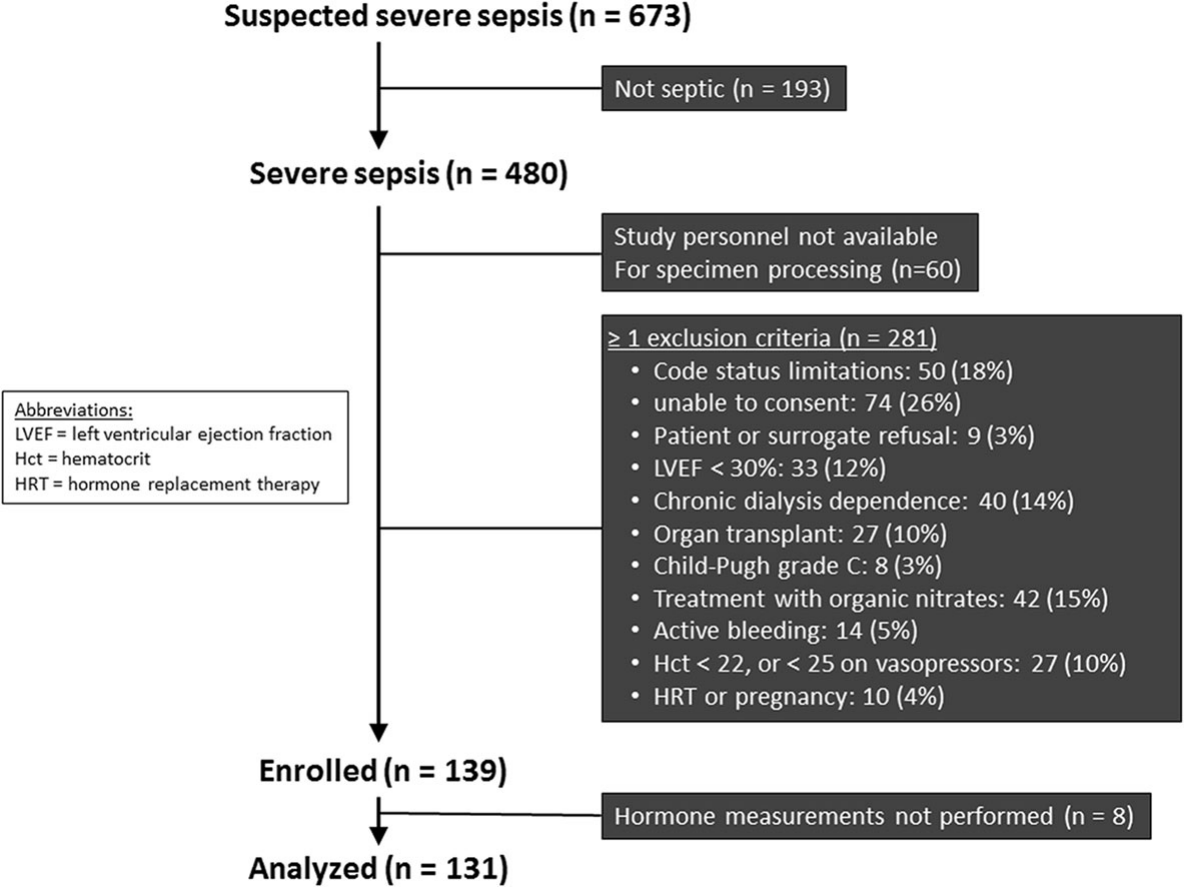
Enrollment algorithm for patients with severe sepsis

This combined cohort and case-control study has been described previously [21–23]. Consecutive patients in the medical or surgical ICU of the University of Rochester Medical Center meeting the 1991 and 2003 consensus conference criteria for severe sepsis [24] were screened for enrollment between 2006 and 2009. The inclusion criteria were (1) having a known or suspected source of infection, (2) meeting two or more systemic inflammatory response syndrome criteria, and (3) having one or more acute organ dysfunctions (for definitions, see Additional file 1: Table S2). Control subjects without acute illness were recruited from the local community and stratified by age and sex to approximate the sepsis cohort. A list of all prospectively defined exclusion criteria is shown in Fig. 1. We also could not enroll eligible patients if research staffing was inadequate to obtain and process blood specimens within 48 h after patients fulfilled severe sepsis criteria. Written informed consent was obtained from all subjects or their surrogates, and the study protocol was approved by the University of Rochester Research Subjects Review Board.

### Measurement of estradiol levels

Venous blood samples were obtained within 48 h of a patient’s fulfilling severe sepsis criteria. A second venous blood sample was collected 72–96 h after the first sample in the remaining patients. Serum samples dedicated to these hormone measurements were stored continuously at −80 °C until analysis. Commercially available kits were used to measure serum concentrations of SHBG (enzyme immunoassay; ALPCO, Salem, NH, USA), estradiol (radioimmunoassay; Diagnostic Systems Laboratories, Webster, TX, USA), and plasma albumin (QuantiChrom™ BCG Albumin Assay Kit; BioAssay Systems, Hayward, CA, USA). All samples were measured in duplicate. The free and albumin-bound serum estradiol concentrations were calculated as previously described (see supplementary methods in Additional file 1) [25–28]. The bioavailable estradiol concentration was calculated as the sum of the free and albumin-bound estradiol concentrations [26, 28]. All laboratory measurements were completed by April 2010.

### Statistical analysis

Estradiol levels had a skewed distribution, so nonparametric Mann-Whitney *U* and Wilcoxon signed-rank tests were used to compare groups. The results are expressed as median (interquartile range). Spearman’s rank correlation coefficients (rho) were calculated between bioavailable estradiol concentration and other continuous severity of illness variables, including Acute Physiology and Chronic Health Evaluation II scores [29], Sepsis-related Organ Failure Assessment (SOFA) scores [30], and the number of organ failure-free days from days 0 to 28 [31].

Multivariable logistic regression was used to assess the independent association between high bioavailable estradiol concentrations (above the median value in sepsis patients) and hospital mortality. Age, sex, and Charlson comorbidity index [32] were clinically judged to be potentially important confounding variables and were therefore included in the base multivariable model. Covariables were sequentially removed if comparisons between the nested model and the base model were insignificant (*p* ≥ 0.10 by likelihood ratio test), beginning with the covariable least associated with the outcome variable and continuing in order until a covariable’s removal caused deterioration in the model fit (*p* < 0.10 by likelihood ratio test). Bioavailable estradiol concentration was then introduced into the parsimonious model. Statistical analyses were performed using SAS version 9.1.3 software (SAS Institute, Cary, NC, USA) and Stata 12 software (StataCorp, College Station, TX, USA). Results were considered statistically significant if *p* ≤ 0.05, unless otherwise specified.

## Results

Between February 2006 and February 2009, 52 healthy volunteers and 139 sepsis patients were enrolled in the study (Fig. 1). Serum sample volumes were insufficient in one healthy volunteer and eight sepsis patients. These subjects were removed from further analysis. At the time of the second hormone measurement, 21 sepsis patients had missing hormone data because of death (*n* = 11), hospital discharge (*n* = 4), or insufficient sample (*n* = 6). Baseline demographics for the healthy volunteer and sepsis patient cohorts are shown in Table 1.

**Table 1:** Clinical characteristics of study subjects

	Controls (*n* = 51)	Sepsis (*n* = 131)	*p* Value	Survivors (*n* = 98)	Nonsurvivors (*n* = 33)	*p* Value
Age, years	60 (53–66)	62 (48–74)	0.65	57 (47–69)	71 (61–78)	<0.01
Male sex	26 (51 %)	75 (57 %)	0.45	56 (57 %)	19 (58 %)	0.97
Race			0.23			0.09
White	48 (94 %)	108 (82 %)		81 (83 %)	27 (82 %)	
African American	3 (6 %)	20 (15 %)		16 (16 %)	4 (12 %)	
Asian	0	1 (1 %)		1 (1 %)	0	
Hispanic/Latino	0	2 (2 %)		0	2 (6 %)	
Charlson comorbidity index [32]	0 (0–1)	3 (1–6)	<0.01	2 (1–5)	4 (3–8)	<0.01
Admission type			–			0.01
Medical patient	–	118 (90 %)		92 (78 %)	26 (22 %)	
Surgical patient	–	13 (10 %)		6 (46 %)	7 (54 %)	
Site of infection			–			0.74
Pulmonary	–	76 (58 %)		54 (55 %)	22 (67 %)	
Intra-abdominal	–	19 (15 %)		14 (14 %)	5 (15 %)	
Urinary	–	15 (11 %)		13 (13 %)	2 (6 %)	
Skin/catheter	–	6 (5 %)		5 (5 %)	1 (3 %)	
Other	–	15 (11 %)		12 (12 %)	3 (9 %)	
Microbiology			–			0.34
Gram-positive bacteria	–	42 (32 %)		30 (31 %)	12 (36 %)	
Gram-negative bacteria	–	20 (15 %)		15 (15 %)	5 (2 %)	
Fungal	–	3 (2 %)		3 (3 %)	0	
Mixed or other	–	27 (21 %)		17 (17 %)	10 (30 %)	
Unknown	–	39 (30 %)		33 (34 %)	6 (2 %)	
Positive blood culture	–	48 (37 %)	–	36 (37 %)	12 (36 %)	0.97
Septic shock^a^	–	107 (82 %)	–	75 (77 %)	32 (97 %)	0.01
APACHE II score	–	24 ± 9	–	22 ± 8	30 ± 9	<0.01
SOFA score^b^						
Minimum	–	7 (5–9)	–	6 (4–8)	11 (8–12)	<0.01
Maximum	–	10 (8–13)	–	9 (8–11)	13 (11–16)	<0.01
Mean	–	8 (6–11)	–	8 (6–9)	12 (9–14)	<0.01
ICU LOS, days	–	6 (3–12)	–	6 (3–11)	8 (3–17)	0.21
Hospital LOS, days	–	15 (9–29)		14 (9–26)	15 (7–39)	0.43
Ventilator-free days 0–28^c^	–	21 (3–28)	–	24 (20–28)	0 (0–0)	<0.01

*Abbreviations: APACHE II* Acute Physiology and Chronic Health Evaluation II [29], *SOFA* Sepsis-related Organ Failure Assessment, *LOS* Length of stay, ICU Intensive care unit

Values are median (interquartile range), number (percentage), or mean ± SD

^a^Shock was defined as hypotension or vasopressor dependence that persisted for ≥3 h despite fluid challenge at the time of diagnosis

^b^Daily SOFA scores were calculated. Values refer to the minimum, maximum, and mean SOFA scores from the day of diagnosis through day 7

^c^Ventilator-free days 0–28 represent the number of days free of ventilator support from the day of diagnosis through day 28

**Fig. 2:**
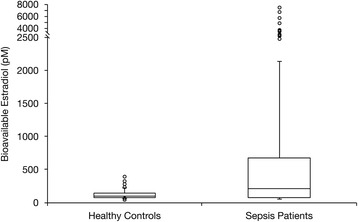
Bioavailable estradiol in sepsis patients versus control subjects. Box plot shows the medians with 25th and 75th percentiles. The *circles* represent outliers beyond the whiskers that designate the 10th and 90th percentiles

Consistent with prior studies, sepsis patients had significantly higher total estradiol concentrations than healthy control subjects (Table 2). Sepsis patients had lower albumin concentrations and higher SHBG concentrations than control subjects. These differences in estradiol carrier proteins would be expected to diminish the bioavailable estradiol fraction in septic patients, consistent with our original hypothesis. Nevertheless, bioavailable estradiol concentrations were still higher in sepsis patients than in control subjects (Table 2 and Fig. 2).

**Table 2:** Estradiol and carrier protein concentrations

	Control subjects vs. sepsis patients			Sepsis, survivors vs. nonsurvivors		
Variable	Controls (*n* = 51)	Sepsis (*n* = 131)	*p* Value	Survivors (*n* = 98)	Nonsurvivors (*n* = 33)	*p* Value
Total estradiol, pM	115 (88–166)	275 (112–916)	<0.01	222 (100–814)	512 (276–1040)	0.01
Albumin-bound estradiol, pM	97 (76–139)	199 (74–637)	<0.01	156 (66–538)	278 (154–855)	0.04
Free estradiol, pM	3 (2–4)	10 (4–46)	<0.01	8 (4–28)	23 (10–73)	<0.01
Bioavailable estradiol, pM	100 (78–142)	211 (78–675)	<0.01	167 (70–566)	312 (164–918)	0.04
Albumin, g/dl	5.24 (4.61–5.71)	2.67 (2.06–3.49)	<0.01	3.00 (2.30–3.74)	2.06 (1.72–2.63)	<0.01
SHBG, nM	9.52 (4.13–22.06)	19.00 (10.50–32.80)	<0.01	19.00 (11.20–32.50)	17.41 (7.92–34.19)	0.70

*SHBG* Sex hormone-binding globulin

Values are given as median (interquartile range)

**Fig. 3:**
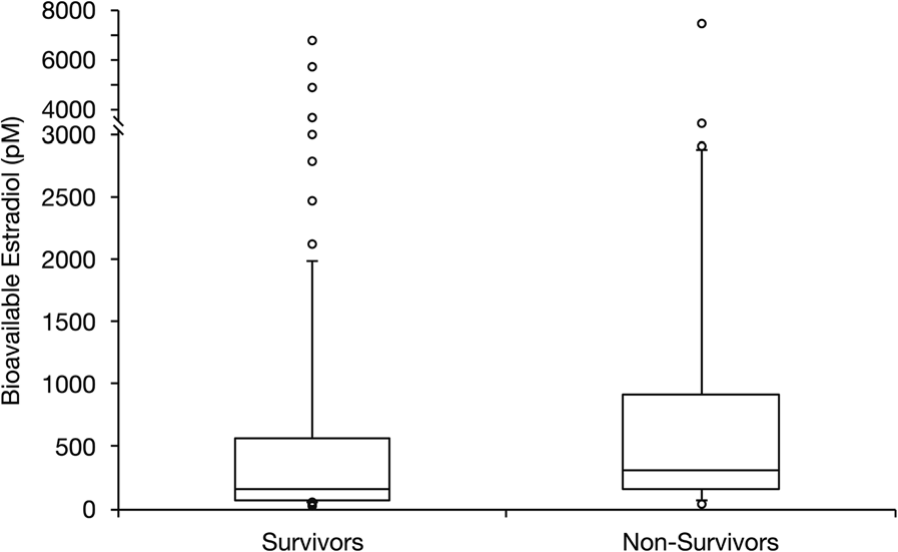
Bioavailable estradiol in sepsis survivors versus nonsurvivors. Box plot shows medians with 25th and 75th percentiles. The *circles* represent outliers beyond the whiskers that designate the 10th and 90th percentiles

Thirty-three (25 %) of the sepsis patients died during hospitalization (see Table 1 for comparison of clinical characteristics between survivors and nonsurvivors). Total and bioavailable estradiol concentrations were significantly higher in nonsurvivors than in survivors (Table 2 and Fig. 3). Albumin was lower among nonsurvivors, while SHBG was similar between the two groups (Table 2). The final multivariable model included age and Charlson comorbidity index. After adjustment for these covariables, patients with higher bioavailable levels had a significantly higher likelihood of dying in the hospital than did those with concentrations less than or equal to the median value (OR 4.27, 95 % CI 1.65–11.06, *p* = 0.003) (Table 3).

**Table 3:** Multivariable logistic regression analysis evaluating the association between bioavailable estradiol and hospital mortality

Dependent variable	Independent variables	Odds ratio^a^ (95 % CI)	*p* Value
Hospital mortality	Bioavailable estradiol ≥ median	4.27 (1.65–11.06)	0.003
	Age in years	1.04 (1.01–1.07)	0.009
	Charlson index ≥ median	4.15 (1.53–11.27)	0.005

The final parsimonious multivariable model included age in years and Charlson comorbidity index dichotomized by the median value (3). This model had good discrimination (c-statistic = 0.73) and acceptable calibration (Hosmer-Lemeshow χ^2^ = 13.4, *p* = 0.10)

^a^Odds ratios refer to the change in likelihood of hospital mortality for patients with bioavailable estradiol concentration ≥211 pM, a 1-year increase in age, and a Charlson comorbidity index ≥3, all after adjustment for the other two variables

Bioavailable estradiol was positively correlated with 7-day maximum and mean SOFA scores (Spearman’s rho = 0.28 and *p* < 0.01 for both) and negatively correlated with the number of organ failure-free days from days 0–28 (Spearman’s rho = −0.26, *p* < 0.01). Bioavailable estradiol concentrations decreased over time in the sepsis patients (see Additional file 1: Table S2). Estradiol concentrations did not differ between men and women (see Additional file 1: Table S3).

## Discussion

The main findings of our study are that bioavailable estradiol levels are elevated in critically ill septic patients compared with healthy controls and in sepsis nonsurvivors compared with survivors. While confirming previous observations, our study uniquely demonstrates that the higher estradiol levels characterizing sepsis are in the biologically active pool and are not an artifact of sepsis-associated alterations in carrier protein concentrations. Furthermore, we found that patients with particularly high bioavailable estradiol levels had a higher risk of death even when we adjusted for potential confounding variables. Finally, higher bioavailable estradiol levels are also linked to higher severity of illness.

We hypothesized that higher bioavailable estradiol concentrations would be associated with lower mortality in septic patients. This hypothesis was driven by laboratory studies suggesting a therapeutic benefit from estrogens. For example, in rodents undergoing cecal ligation and puncture, mortality was lower in proestrus females than in ovariectomized females or in males [6]. Furthermore, treatment with an estrogen receptor agonist improved survival in this animal model [7]. Our contradictory findings highlight the limitations of observational clinical research: It is capable of detecting clinical associations but incapable of demonstrating causality or pathophysiological significance. In this case, for example, higher bioavailable estradiol levels in sepsis nonsurvivors do not necessarily indicate a deleterious excess. If estradiol has beneficial biological effects, our findings could represent an adaptive but insufficient response to sepsis, diminished receptor sensitivity, or both. Alternatively, bioavailable estradiol may simply be an acute-phase reactant without pathophysiological significance. Finally, it is possible that bioavailable estradiol has adverse pathological effects, with higher levels promoting adverse sepsis outcomes. Unfortunately, our findings do not allow us to draw any inference about which of these three possibilities is operative.

### Comparison with previous literature

Several studies have shown that elevated total estradiol levels are linked to mortality in both noninfected and infected critically ill patients [12–14, 33–35]. Our findings are consistent with these prior results and extend them by demonstrating that the bioavailable estradiol fraction is also elevated in sepsis and sepsis nonsurvivors. By calculating the bioavailable estradiol concentration, we found that associations between estradiol and sepsis, and between estradiol and sepsis mortality, persist after controlling for the changes in carrier proteins that occur in critical illness. To our knowledge, this has not been reported previously.

### The influence of sex

We found that bioavailable estradiol concentration did not vary by sex. These findings are consistent with previous reports that total estradiol did not significantly differ between critically ill men and women [12–14, 33, 36]. Despite these similarities in hormone levels, recent, large sepsis epidemiological studies indicate that sepsis mortality varies by sex [37–39]. Together, these results suggest that sex differences in mortality are not mediated by hormonal effects. Other possible mechanisms need to be considered, including sex differences in care delivery [2–4].

### Strengths and limitations

The strengths of our study are its prospective design and inclusion of the measurements necessary for calculation of bioavailable estradiol. Our study has several limitations. First, its observational design precludes establishment of a causal relationship between bioavailable estradiol concentrations and sepsis or mortality. Second, we were unable to investigate relationships between estradiol, inflammation, and physiological stress because we did not measure markers of the inflammatory and stress responses. Third, our samples were obtained a median of 27 h after patients met diagnostic criteria, and the results may have been different if samples had been obtained earlier. Third, we cannot determine if our findings are unique to sepsis or characterize critical illness more generally. Finally, our inclusion criteria were based on prior sepsis syndrome definitions that have since been updated [40]. Nevertheless, all patients had known or suspected infection and SOFA scores >2 (Table 1), indicating that they also satisfied the latest revised sepsis criteria.

## Conclusions

The serum bioavailable estradiol concentration is significantly elevated in critically ill septic patients compared with healthy control subjects of similar age and sex distribution. Further, high bioavailable estradiol levels independently predict mortality in sepsis. Bioavailable estradiol levels were similar in septic men and women. Our results do not provide an explanation for previously documented sex differences in sepsis outcomes, and more research is warranted to explore these differences. Additional study is also required to establish whether estradiol’s predominant effects in sepsis are deleterious, beneficial, or neutral.

## Key messages

- The bioavailable fraction of circulating estradiol is high in sepsis and is not affected by sex.
- Nonsurvivors have higher bioavailable estradiol levels than survivors, and higher levels are independently associated with hospital mortality.

## Additional file

Additional file 1: Table S1. Inclusion criteria. **Table S2.** Measurement 1 vs. measurement 2 in survivors and nonsurvivors. **Table S3.** Concentrations of estradiol and carrier proteins by gender. (DOCX 35 kb)

## Abbreviations

APACHE: Acute Physiology and Chronic Health Evaluation
ICU: Intensive care unit
LOS: Length of stay
SHBG: Sex hormone-binding globulin
SOFA: Sepsis-related Organ Failure Assessment
