# Supplementary material for: Bioavailable estradiol concentrations are elevated and predict mortality in septic patients: a prospective cohort study
Source: Crit Care. 2016 Oct 21;20:335. doi: 10.1186/s13054-016-1525-9 (PMC5073735; doi:10.1186/s13054-016-1525-9)
Supplement: Additional file 1: Table S1. — Inclusion criteria. Table S2. Measurement 1 vs. measurement 2 in survivors and nonsurvivors. Table S3. Concentrations of estradiol and carrier proteins by gender. (DOCX 35 kb) [file 13054_2016_1525_MOESM1_ESM.docx]

**Supplemental Methods:**

***Inclusion criteria for enrollment***

Table S1. Inclusion criteria (must meet all three of the enrollment criteria)

| **Enrollment Criteria** | **Description** |
| --- | --- |
| 1. Known or suspected infection | ≥ one of the following^a^:   - Infection documented in the medical record by the critical care physician providers - WBCs isolated from a normally sterile body fluid - Perforated viscus - Radiographic evidence of pneumonia together with purulent sputum - Syndromes associated with infection (e.g., ascending cholangitis, cellulitis). |
| 1. SIRS components | ≥ two of the following^b^:   - T > 38°C, or < 36 °C - HR > 90 bpm - RR > 20 or pCO_2_ < 32 - WBC > 12k or < 4k, or >10% bands |
| 1. Organ dysfunctions | ≥ one of the following^a^:   - Cardiovascular: SBP < 90 mm Hg, MAP < 60 mm Hg, vasopressor requirement, or clinical evident of hypoperfusion - Respiratory: P:F ratio < 250, or < 200 if lungs are the only dysfunctional organ - Renal: urine output < 0.5 mL/kg/hour despite fluid resuscitation - Acid-base: metabolic acidosis and lactate concentration > 2 mM - Neurologic: altered mental status without other cause - Hematologic: Platelet count < 80,000 or > 50% decreased from baseline |

Definition of abbreviations: SIRS = systemic inflammatory response syndrome; T = temperature; HR = heart rate; RR = respiratory rate; WBC = white blood cell count; k = thousand cells/ mm^3^; SBP = systolic blood pressure; MAP = mean arterial pressure; P:F ratio = partial pressure of arterial oxygen : fraction of inspired oxygen ratio.

^a^adapted from Bernard et al [[1](#_ENREF_1)]

^b^as defined by Bone et al [[2](#_ENREF_2)]

Free estradiol (FE) levels were calculated from the measured concentrations of albumin (A), total estradiol (TE), and sex hormone binding globulin (SHBG), according to the equation [[3-5](#_ENREF_3)]:

$FE= \frac{TE-(N \times FE)}{(K_{S} \left[ SHBG-TE+N\times FE \right]}$ *(Equation 1)*

where K_S_ = the affinity constant of SHBG for estradiol (3.1 x 10^8^ L/mol) and

$N= K_{a}\times A+1$,

where K_a_ = the affinity constant of A for estradiol (4.2 x 10^4^ L/mol).

Solving for FE yields the following second-degree equation [[4](#_ENREF_4)]:

$FE= \frac{\left( - \left[ K_{S}\times SHBG \right]- \left[ K_{S}\times TE \right]+N \right) \pm\sqrt{\left( \left[ \left\{ K_{S}\times SHBG \right\}- \left\{ K_{S}\times TE \right\}+N \right]^{2}+ \left[ 4\times\left\{ K_{S} \times N \right\}\times\left\{ -TE \right\} \right] \right)}}{\left. 2\times\left( K_{S}\times N \right) \right.}$ *(Equation 2).*

Equation 2 has the form:

$$x= \frac{-b \pm\sqrt{b^{2}-4ac}}{2a}$$

This allows calculation of FE using a computerized quadratic equation solver [[6](#_ENREF_6)].

The albumin-bound estradiol (AE) concentration was calculated from the following equation [[7](#_ENREF_7)]:

$$AE= K_{a}\times A\times FE$$

Finally, the bioavailable estradiol concentration was calculated as the sum of FE and AE [[3](#_ENREF_3), [7](#_ENREF_7)].

**References:**

1. Bernard GR, Vincent JL, Laterre PF, LaRosa SP, Dhainaut JF, Lopez-Rodriguez A, Steinbrub JS, Garber GE, Helterbrand JD, Ely W, Fisher CJ: **Efficacy and safety of recombinant human activated protein C for severe sepsis**. *N Engl J Med* 2001, **344**:699-709.

2. Bone RC, Balk RA, Cerra FB, Dellinger RP, Fein AM, Knaus WA, Schein RM, Sibbald WJ: **Definitions for sepsis and organ failure and guidelines for the use of innovative therapies in sepsis**. *Chest* 1992, **101**:1644-1655.

3. Rinaldi S, Geay A, Dechaud H, Biessy C, Zeleniuch-Jacquotte A, Akhmedkhanov A, Shore RE, Riboli E, Toniolo P, Kaaks R: **Validity of free testosterone and free estradiol determinations in serum samples from postmenopausal women by theoretical calculations**. *Cancer Epidemiol Biomarkers Prev* 2002, **11**(10 Pt 1):1065-1071.

4. Svartberg J, Midtby M, Bonaa KH, Sundsfjord J, Joakimsen RM, Jorde R: **The associations of age, lifestyle factors and chronic disease with testosterone in men: the Tromso Study**. *Eur J Endocrinol* 2003, **149**(2):145-152.

5. Bjornerem A, Straume B, Midtby M, Vonnebo V, Sundsfjord J, Svartberg J, Acharya G, Oian P, Berntsen GKR: **Endogenous sex hormones in relation to age, sex, lifestyle factors, and chronic diseases in a general population: the Tromso study**. *J Clin Endocrinol Metab* 2004, **89**:6039-6047.

6. **arachnoid.com** [[www.arachnoid.com/quadsolver/index.html](http://www.arachnoid.com/quadsolver/index.html)]

7. **Free estradiol and breast cancer risk in postmenopausal women: comparison of measured and calculated values**. *Cancer Epidemiol Biomarkers Prev* 2003, **12**(12):1457-1461.

**Supplemental Results**

Table S2. Measurement 1 vs. Measurement 2 in survivors and non-survivors *^a^*

|  | | Hospital survivors (n = 90) | | |  | | Hospital non-survivors (n = 20) | | |
| --- | --- | --- | --- | --- | --- | --- | --- | --- | --- |
| Variable | M 1 | | M 2 | *p* value | |  | M 1 | M 2 | *p* value |
| Bioavailable estradiol (pM) | 166 (70 – 675) | | 80 (50 – 177) | < 0.01 | |  | 236 (140 – 567) | 107 (66 – 694) | < 0.01 |

*^a^* Values refer to median (interquartile range).

Table S3. Concentrations of estradiol and carrier proteins by gender

| **Estradiol/ carrier protein** | **Male** | **Female** | ***p*** |
| --- | --- | --- | --- |
| Total estradiol (nM)  Healthy controls  Severe sepsis patients | 0.116 [0.090-0.207], n=26  0.257 [0.106-0.814], n=75 | 0.105 [0.073-0.142], n=25  0.306 [0.125-0.982], n=56 | 0.38  0.44 |
| Albumin-bound estradiol (nM)  Healthy controls  Severe sepsis patients | 0.097 [0.085-0.138], n=26  0.194 [0.073-0.565], n=75 | 0.094 [0.070-0.139], n=25  0.211 [0.079-0.756], n=56 | 0.84  0.79 |
| Free estradiol (nM)  Healthy controls  Severe sepsis patients | 0.003 [0.002-0.004], n=26  0.010 [0.004-0.045], n=75 | 0.003 [0.003-0.004], n=25  0.011 [0.004-0.048], n=56 | 0.38  0.75 |
| Bioavailable estradiol (nM)  Healthy controls  Severe sepsis patients | 0.101 [0.087-0.142], n=26  0.202 [0.077-0.614], n=75 | 0.097 [0.073-0.142], n=25  0.219 [0.082-0.805], n=56 | 0.82  0.79 |
| Albumin (g/dL)  Healthy controls  Severe sepsis patients | 8.28 [7.28-8.79], n=26  4.03 [2.99-5.67], n=75 | 7.57 [6.37-8.21], n=25  4.02 [3.46-4.87], n=56 | 0.05  0.93 |
| SHBG (nM)  Healthy controls  Severe sepsis patients | 12.26 [6.21-27.83], n=26  14.99 [6.32-24.74], n=75 | 7.16 [2.41-12.44], n=25  25.00 [14.95-37.46], n=56 | 0.01  0.00 |
